# Effect of Oral High Molecular Weight Hyaluronic Acid (HMWHA), Alpha Lipoic Acid (ALA), Magnesium, Vitamin B6 and Vitamin D Supplementation in Pregnant Women: A Retrospective Observational Pilot Study

**DOI:** 10.3390/clinpract13050100

**Published:** 2023-09-15

**Authors:** Eligio Parente, Giulia Colannino, Gabriele Bilotta, Maria Salomé Bezerra Espinola, Sara Proietti, Mario Montanino Oliva, Isabella Neri, Cesare Aragona, Vittorio Unfer

**Affiliations:** 1 Clinica Villa delle Querce, 80136 Naples, Italy; 2 Alma Res Fertility Center, 00198 Rome, Italy; 3 Systems Biology Group Lab., 00161 Rome, Italy; 4 R&D Department Lo.Li Pharma srl, 00156, Rome, Italy; 5 Department of Obstetrics and Gynecology, Santo Spirito Hospital, 00193 Rome, Italy; 6 Obstetrics Unit, Mother Infant Department, University Hospital Policlinico of Modena, 41124 Modena, Italy; 7 UniCamillus-Saint Camillus International University of Health and Medical Sciences, 00131 Rome, Italy

**Keywords:** high hyaluronic acid (HMWHA), pregnant women, prevention of adverse events, oral treatment

## Abstract

Background—Pregnancy represents a nutritional challenge, since macro- and micronutrients intake can affect mother’ health and influence negative outcomes. The aim of this retrospective pilot study is to evidence whether the oral supplementation with high molecular weight hyaluronic acid (HMWHA), in association with alpha lipoic acid (ALA), magnesium, vitamin B6 and vitamin D, in pregnant women, could reduce adverse effects, such as PTB, pelvic pain, contraction and hospitalization. Methods—Data were collected from *n* = 200 women treated daily with oral supplements of 200 mg HMWHA, 100 mg ALA, 450 mg magnesium, 2.6 mg vitamin B6 and 50 mcg vitamin D (treatment group) and from *n* = 50 women taking with oral supplements of 400 mg magnesium (control group). In both groups, supplementation started from the 7th gestational week until delivery. Results—Oral treatment with HMWHA, in association with ALA, magnesium, vitamin B6 and vitamin D in pregnant women, significantly reduced adverse events, such as PTB (*p* < 0.01), pelvic pain and contractions (*p* < 0.0001), miscarriages (*p* < 0.05) and admission to ER/hospitalization (*p* < 0.0001) compared with the control group. Conclusions—Despite HMWHA having been poorly used as a food supplement in pregnant women, our results open a reassuring scenario regarding its oral administration during pregnancy.

## 1. Introduction

Nutrition plays an important role in supporting physiological pregnancy by providing the necessary nutrients for fetal growth and decreasing the risk of negative outcomes, such as preterm delivery, spontaneous abortion [1,2] or maternal hypertensive disorders [3,4].

Therefore, several nutrients are recommended for dietary supplementation in pregnant women; among them, alpha lipoic acid (ALA), magnesium, vitamin B6 and vitamin D play a pivotal role in maintaining physiological pregnancy [5,6,7].

ALA is a natural antioxidant, anti-inflammatory and immunomodulatory molecule, which decreases the secretion of inflammatory cytokines (TNF-α, IL-1β and IL-17) and stimulates the release of the anti-inflammatory cytokine IL-10, thus reducing the risk of miscarriage in the first trimester of pregnancy [8]. In patients at risk of spontaneous abortion, ALA administration (600 mg/day) is efficient in accelerating the resorption of subchorionic hematoma and improving clinical symptoms [9,10]. Moreover, ALA may protect against premature cervical shortening, thus reducing the risk of preterm labor onset and symptomatology [11].

Vitamin B6 is a water-soluble compound that generally acts as coenzyme in different metabolic reactions. It has a crucial role in the physiologic development and function, and it is widely used as a supplement during pregnancy due to its beneficial effects [12]. It preserves nerve function, counteracts tiredness and fatigue, sustains the functioning of the immune system, regulates hormonal activity and psychological functions [13] and, moreover, reduces nausea/vomiting in pregnancy [14]. Vitamin D is a fat-soluble hormone with progesterone-like activity that plays a pivotal role in calcium, magnesium and phosphate homeostasis and acts as an antiproliferative/immunomodulatory mediator preserving uterine quiescence [15]. Scientific studies highlighted an association between adequate levels of vitamin D during pregnancy and favorable maternal and fetal–neonatal outcomes [16]. Vitamin D deficiency in pregnancy is very common and is related to preeclampsia, gestational diabetes, disorders in bone formation and PTB risk [17]. A recent systematic review and meta-analysis has also demonstrated that women deficient in vitamin D are at significantly increased risk of miscarriage compared with those who are not [18].

Magnesium is one of the ten essential metals in humans and its supplementation during pregnancy correlates with a reduced risk of fetal growth restriction and preeclampsia [19].

Another interesting natural molecule is hyaluronic acid (HA). HA is widely used in several branches of medicine (pulmonology, orthopedics, esthetic medicine, gynecology, ophthalmology etc.) without contraindications or reported interactions with other drugs [20,21]. HA is a fundamental component of extracellular matrix (ECM) and is found in the fluids and tissues of the reproductive system of humans, the stromal structures of the uterus and placenta; the angiogenic regions of decidua basalis; cumulus cells; cervical mucus; and oviductal, uterine and follicular fluid [22,23]. HA is a very peculiar molecule because it has multiple functions depending on its molecular weight [24]. Specifically, high molecular weight hyaluronic acid (HMWHA) physiologically facilitates the attachment of the embryo before implantation, interacting with CD44 and upregulating osteopontin, another ligand of CD44 and an adhesive molecule upregulated by progesterone [25]. Animal models recently demonstrated that HMWHA significantly counteracts the effects of mifepristone and PGE2 on PTB induction in female albino Wistar rats by delaying the delivery time and reversing the upregulation of pro-inflammatory cytokines in uterine tissues [26].

Moreover, several studies reported that the supplementation of media culture with HMWHA in ART procedures improves blastocyst adhesion, embryo development and viability in both human and animal models. Hyaluronic acid can successfully replace albumin as a human embryo transfer medium, resulting in comparably high pregnancy and implantation rates [27]. Overall, clinical pregnancy (CPRs) and implantation rates (IRs) significantly increased with the use of hyaluronan-enriched transfer medium (HETM); this was most evident in women > 35 years of age, in women who had only poor-quality embryos available for transfer and in women who had previous implantation failures [28].

A recent preliminary study in 53 pregnant women with threatened miscarriage evaluated whether the oral intake of HMWHA, in combination with ALA, magnesium, vitamin B6 and vitamin D, can improve the therapy with vaginal progesterone (P4) in the resorption of subchorionic hematoma. Results demonstrated that subjective symptoms, such as abdominal pain/uterine contractions, as well as vaginal bleeding, showed a faster decrease in cases and a faster resorption of the subchorionic hematoma than in controls after 7 days and 14 days of treatment [29]. Despite all this pre-clinical and clinical evidence regarding the supportive role of HMWHA in female reproductive biology [30,31], its use as an oral supplement during gestation has been poorly evaluated.

Therefore, the aim of this pilot study is to examine whether HMWHA, in association with ALA, magnesium, vitamin B6 and vitamin D, could support physiological pregnancy by preventing and reducing adverse events such as miscarriage, preterm birth (PTB), uterine contractions and placental abruption in pregnant women.

## 2. Materials and Methods

This is an observational retrospective pilot study in which we collected data from hospital logbooks about a total of 250 pregnant women aged between 25–41 years old, at the 7th gestational week, from April 2021 to April 2022. Key exclusion criteria included the presence of relevant concomitant pathologies, such as vaginitis, HPV infection, thyroid disorders, arterial hypertension, diabetes, PCOS, preeclampsia, maternal autoimmune diseases, antiphospholipid syndrome, lupus, hepatitis, thalassemia, HIV/AIDS, cancer or a diagnosed alcohol or drug addiction. Data were collected from: (1) a total of *n* = 200 pregnant women were given oral supplement tablets containing 200 mg high molecular weight hyaluronic acid (HMWHA) in combination with 100 mg alpha-lipoic acid (ALA), 450 mg magnesium, 2.6 mg vitamin B6 and 50 mcg vitamin D (2 tablets/die of DAV^®^-HA, Lo.Li. Pharma srl, Rome, Italy) (treatment group) and (2) a total of *n* = 50 pregnant women were given oral supplements of 400 mg magnesium (twice a day) (control group). Both treatments started from the 7th gestational week until delivery. All patients provided written informed consent for their data to be used. All the collected retrospective data were de-identified prior to access by the authors in accordance with the Good Clinical Practice guidelines and the Declaration of Helsinki. Ethical notification: CE Alma Res 014/2023.

### Statistical Analysis

Data analysis was performed using SAS^®^ (Version 9.4, SAS Institute Inc., Cary, NC, USA). The level of statistical significance was set at *p* < 0.05. Fisher’s exact test and the chi-square test were applied to determine the significant difference between categorical variables. Fisher’s exact test was applied when the expected counts were less than 5.

## 3. Results

Patients in both groups were similar in terms of baseline characteristics: aged between 25 and 41 years, 7th gestational week, absence of relevant concomitant pathologies (such as vaginitis, HPV infection, thyroid disorders, arterial hypertension, diabetes, PCOS, preeclampsia, maternal autoimmune diseases, antiphospholipid syndrome, lupus, hepatitis, thalassemia, HIV/AIDS, cancer or a diagnosed alcohol or drug addiction), and no previous miscarriages. Record analyses highlighted that patients were constantly monitored and examined every four weeks until delivery. The percentage of adverse events, such as preterm birth (PTB) (*p* < 0.01), pelvic pain, spontaneous contractions (*p* < 0.0001), miscarriage (*p* < 0.05) and admission to ER/hospitalization (*p* < 0.0001) were significantly lower in the treatment group compared with the control group (Table 1). The number of cases of placental abruption did not differ between groups.

## 4. Discussion

Our results demonstrate for the first time that oral HMWHA in combination with ALA, magnesium, vitamin B6 and vitamin D could counteract adverse events in pregnancy, such as PTB, spontaneous contractions, miscarriages and hospitalization.

ALA is totally safe in pregnant women [32] and it exerts antioxidant, anti-inflammatory and immunomodulatory activities by normalizing effect against the alterations of the cervix and vaginal tissues that occur during PTB [33]. ALA supplementation significantly reduces the incidence of spontaneous contractions [32] by decreasing the expression of two enzymes involved in PTB, nuclear factor-kappa B (NF-KB) [34] and metalloproteinase-9 (MMP-9) [35]; moreover, in association with P4, ALA contributes to the faster healing of subchorionic hematomas in patients with threatened miscarriage in comparison to the standard progesterone approach [9]. Moreover, ALA, in association with magnesium, vitamin B6 and vitamin D, significantly preserves the cervical length between the first and the second trimester of gestation in pregnant women, presenting risk factors for PTB and reducing hospital admissions for threatened PTB, as compared with the control group [36].

Despite its role in female reproductive biology and scientific evidence regarding its supportive role during gestation [30], oral HMWHA has been poorly investigated as a food supplement in pregnant women. The most recent conference proceeding states that HMWHA, in combination with ALA, magnesium, vitamin B6 and vitamin D, can improve therapy with vaginal progesterone (P4) for the resorption of subchorionic hematoma. Results demonstrate that subjective symptoms, such as abdominal pain/uterine contractions, as well as vaginal bleeding, showed a faster decrease in cases and a faster resorption of the subchorionic hematoma than in controls after 7 days and 14 days of treatment [29].

Several scientific studies support the hypothesis that HMWHA contributes to preserving physiological pregnancy by supporting progesterone (P4) activity [30]. Endogenous P4, indeed, is crucial not only for establishing [37] but also for maintaining pregnancy, since it hinders pro-inflammatory cytokine production, thus suppressing myometrial contractility and preserving uterine quiescence until labor [38]. Our results demonstrate that pelvic pain and spontaneous contractions were significantly decreased in the treatment group (1.5%) compared with the control group (30%) (*p* < 0.0001). Because maintaining uterine relaxation is one of “strategies” to avoid PTB, it is not surprising that in the treatment group, the percentage of PTB is significantly lower compared to the control group (1.5% versus 10%) (*p* < 0.01).

HMWHA may also support P4 activity by increasing Progesterone Receptor Membrane Component 1 (PGRMC1) expression, thus preserving uterine quiescence until labor [39]. It is not by chance that in the treatment group with HMWHA supplementation, pelvic pain and spontaneous contractions are significantly reduced compared with the control group (1.5% vs. 30%; *p* < 0.0001). In general, endogenous P4 seems to regulate myometrial contractility both through genomic and non-genomic pathways [40,41]. In vitro contractility studies demonstrated that PGRMC1 is highly expressed at the maternal–fetal interface and may mediate the activity of P4 on the myometrium during pregnancy [42]. PGRMC1 is a non-classical progesterone receptor that is upregulated during pregnancy and downregulated near delivery [42] or in pathological gestational conditions [43,44] by inducing fetal membrane dysfunctions that promote their premature rupture [45]. The biological significance of PGRMC1 is strengthened by the fact that PGRMC1 expression is upregulated in pregnant and downregulated during term and preterm labor, as demonstrated in a recent work in which PGRMC1 expression was decreased in women who suffered from recurrent miscarriages (RM) and PTB [45,46]. Experiments demonstrated that HMWHA also preserves the immunotolerance mechanisms of early pregnancy by inducing the secretion of anti-inflammatory cytokines, such as IL-10, and inhibiting the expression of pro-inflammatory factors, such as tumor necrosis factor alpha (TNF-α) or interferon gamma (IFN-γ) [47]. Moreover, HMWHA stimulates immunomodulation at the maternal–fetal interface by promoting the differentiation of T-naïve into Treg cells [48], which helps to maintain immune tolerance and homeostasis as well as the polarization of decidual macrophages into immunosuppressive M2 [49]. When the endogenous content of HMWHA is reduced, immune imbalance at the maternal–fetal interface occurs and impairs pregnancy, as observed in women who suffer from recurrent miscarriages [49,50]. HMWHA is also essential for the regulation of Toll-like receptor (TLR) signaling pathways; in fact, it shields TLR2 and TLR4 receptors by covering their surfaces as a protective barrier, avoiding the pro-inflammatory stimulations that may induce PTB [51].

## 5. Conclusions

Our observational retrospective study investigated the effect of oral HMWHA supplementation in association with ALA, magnesium, vitamin B6 and vitamin D in pregnant women. Undoubtedly, future studies, i.e., randomized control studies with larger samples, are necessary to deepen our preliminary results. Although we did not record long-term data on the patients’ (mother and infant) outcomes, currently there is no reported scientific evidence showing harmful effects or contraindications regarding the administration of the studied supplements during gestation.

## Figures and Tables

**Table 1:** Pregnancy outcomes. Dichotomous variables are reported as numbers and ratios (%). Significant differences (*p* < 0.05) between the two groups are indicated in bold. Abbreviations = preterm birth (PTB); emergency room (ER).

Pregnancy Outcomes	Treatment Group *n* = 200 pz	Control Group *n* = 50 pz	*p*-Value
Placental abruption	2 (1%)	0	*p* = 1.0000
PTB	3 (1.5%)	5 (10%)	*p* < 0.01
Pelvic pain and spontaneous contractions	3 (1.5%)	15 (30%)	*p* < 0.0001
Miscarriages	0	2 (4%)	*p* < 0.05
Access to ER/hospitalization	1 (0.5%)	15 (30%)	*p* < 0.0001

## Data Availability

The data presented in this study are available on request from the corresponding author.
